# Postoperative Outcomes of Star Plate Fixation Versus Tension Band Wiring in Displaced Transverse Patella Fractures

**DOI:** 10.7759/cureus.103443

**Published:** 2026-02-11

**Authors:** Amandeep S Bakshi, Debjit Banerjee, Jaspreet Singh, Sudhanshu Arya, Mukul Sharma

**Affiliations:** 1 Orthopedics, Government Medical College &amp; Hospital and Rajindra Hospital, Patiala, IND; 2 Orthopedic Surgery, Government Medical College &amp; Hospital and Rajindra Hospital, Patiala, IND

**Keywords:** bostman's score, patella fracture, star plate, tbw, union

## Abstract

Background: Patellar fractures may occur due to either direct trauma or indirect forces, with the mechanism of injury largely dictating the fracture pattern. Hence, the present study was conducted to compare the efficacy of tension band wiring (TBW) and star plate patella among patients with patella fractures.

Materials and methods: This prospective study compared TBW and star plate fixation in 40 patients with displaced transverse patellar fractures, divided equally into two groups. Standardized preoperative assessment, surgical protocols, and postoperative rehabilitation were followed, with outcomes assessed clinically and radiologically over six months using the Böstman scoring system.

Results: Both groups showed comparable fracture union rates (100% in TBW vs. 95% in star plate) and mean union times (10.9 vs. 11.9 weeks, p = 0.338). Functional outcomes were similar, with excellent-to-good results in all TBW cases and 90% of star plate cases (p = 0.8116). Complications were slightly higher in the star plate group, including one non-union, more infections, and stiffness.

Conclusion: Both TBW and star plate fixation achieved comparable union rates, union times, and functional outcomes in displaced transverse patellar fractures. However, the star plate group showed a slightly higher complication rate, including one case of non-union.

## Introduction

The patella is the largest sesamoid bone in the body and is a key component of the knee extensor apparatus. By increasing the effective lever arm of the quadriceps muscle, it improves the efficiency of knee extension while simultaneously shielding the femoral condyles and transmitting forces from the quadriceps to the tibia through the patellar tendon. Loss of patellar integrity can therefore result in pain, weakness, and substantial functional limitation if appropriate treatment is not instituted in a timely manner [1].

Patellar fractures account for approximately 0.5%-1% of all skeletal injuries and are most frequently observed in physically active adults, with a higher prevalence in men due to greater exposure to high-energy trauma [2]. The mechanism of injury plays a decisive role in determining fracture pattern, displacement, and degree of comminution [3,4]. Indirect injury, which occurs more commonly, results from sudden eccentric contraction of the quadriceps muscle against a flexed knee, typically producing transverse fracture configurations due to tensile overload of the extensor mechanism [3,4]. As a biomechanical fulcrum, the patella amplifies quadriceps efficiency and is subjected to considerable forces during routine activities. Consequently, disruption of this structure can markedly impair knee function, emphasizing the importance of anatomical restoration and early mobilization in achieving favorable postoperative outcomes [4,5].

In contrast, direct trauma-such as falls directly onto the knee, dashboard injuries during road traffic accidents, or sports-related impacts-often results in comminuted or stellate fracture patterns and is frequently associated with damage to the articular cartilage and surrounding soft tissues [5]. Overall, patellar fractures constitute nearly 1% of all skeletal fractures and present a distinct clinical challenge due to the patella’s critical role in maintaining extensor mechanism continuity and patellofemoral joint congruence [5]. Open patellar fractures represent approximately 6%-9% of cases and are usually linked to high-energy mechanisms with associated musculoskeletal or systemic injuries [6].

Fracture classification systems, including the Arbeitsgemeinschaft für Osteosynthesefragen/Orthopaedic Trauma Association (AO/OTA) classification, provide a standardized framework for describing fracture morphology and assist in guiding management decisions [7-9]. Non-displaced fractures with preserved extensor function can often be managed non-operatively, whereas displaced fractures or those associated with disruption of the extensor mechanism generally require surgical intervention to restore extensor continuity and articular alignment [10].

Tension band wiring (TBW) is based on the biomechanical principle of converting tensile forces acting across the anterior patella into compressive forces at the fracture site and has long been considered the standard operative technique for transverse patellar fractures [11,12]. While TBW has demonstrated reliable outcomes in simple fracture patterns, reported complications include implant migration, symptomatic hardware prominence, loss of reduction, and the need for secondary procedures for implant removal, all of which may negatively influence functional recovery [13]. These shortcomings are particularly pronounced in comminuted fractures and osteoporotic bone, where achieving stable fixation can be challenging.

Recent advances in implant design have led to the development of low-profile locking plate systems, including star-shaped and variable-angle patellar plates, which allow multidirectional screw placement and improved control of fracture fragments. Biomechanical studies have shown that locked plating constructs provide greater stability and resistance to displacement than TBW in complex fracture patterns [13-15]. Clinical evidence has further suggested that locking plate fixation may facilitate earlier rehabilitation, reduce implant-related irritation, and result in satisfactory functional outcomes, especially in comminuted fractures.

With the growing use of star plate fixation in the management of patellar fractures, a direct comparison of postoperative functional outcomes between locking plate constructs and conventional TBW is warranted. With the introduction of various government schemes, an expensive star plate is available free of cost to the general population, resulting in high demand in the near future. Assessment of parameters such as knee range of motion, extensor strength, pain, complication rates, and patient-reported outcome measures is essential to determine the most appropriate fixation strategy for achieving durable functional recovery following patellar fracture fixation.

## Materials and methods

Study design and setting

**Figure 1:**
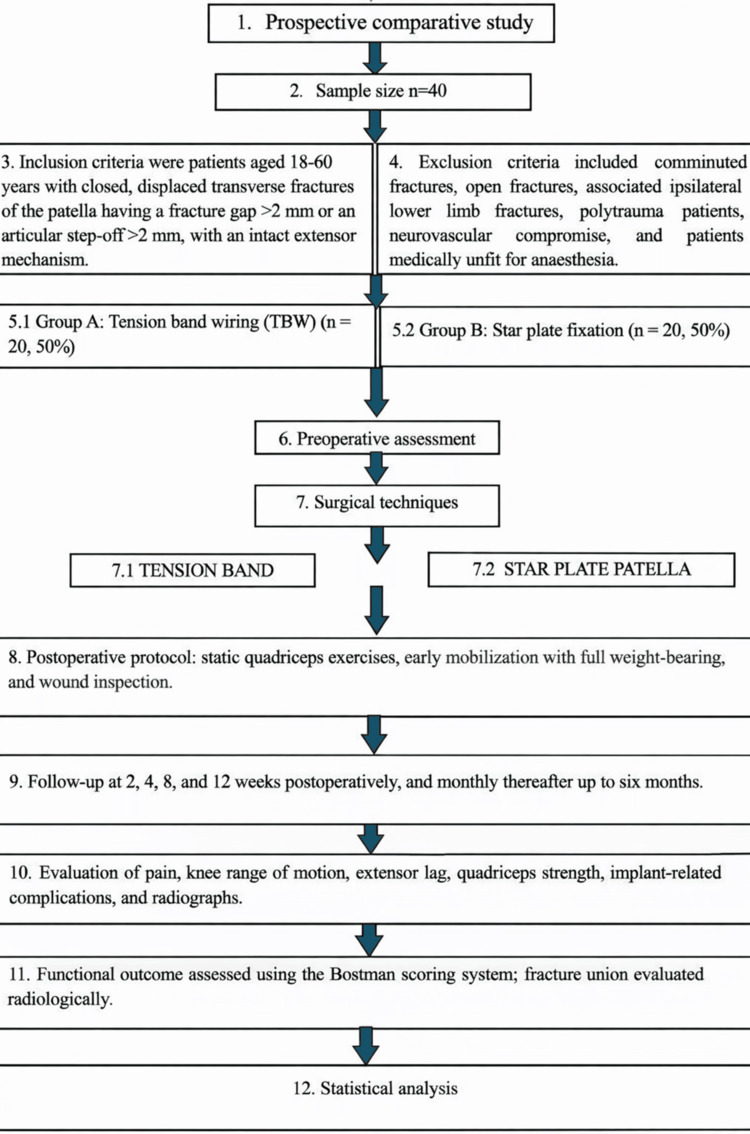
Flowchart diagram describing the study methodology.

This prospective comparative study was carried out at a tertiary care orthopedic institution (Government Medical College and Rajindra Hospital, Patiala, Punjab, India) and included skeletally mature patients diagnosed with displaced transverse fractures of the patella. A total of 40 patients were enrolled after obtaining approval from the institutional ethics committee, and written informed consent was secured from all participants prior to inclusion in the study (Figure 1).

Study population

The study comprised 40 patients between 18 and 60 years of age who sustained displaced transverse patellar fractures. Based on the fixation technique employed, patients were equally assigned to two treatment groups: Group A: TBW (n = 20) and Group B: star plate fixation (n = 20).

Inclusion and exclusion criteria

Patients were eligible for inclusion if they were aged 18-60 years and presented with closed transverse fractures of the patella showing displacement, defined as a fracture gap greater than 2 mm or an articular step-off exceeding 2 mm, with preservation of extensor mechanism continuity. Patients were excluded if they had comminuted fracture patterns, open injuries, associated fractures of the ipsilateral lower limb, polytrauma, neurovascular deficits, or medical conditions that rendered them unfit for anesthesia.

Preoperative evaluation

All patients underwent a comprehensive preoperative assessment, including documentation of the mechanism and time interval from injury. Clinical evaluation focused on assessing knee swelling, localized tenderness, active and passive range of motion, and extensor mechanism integrity. Standard radiographic imaging of the knee was performed for fracture characterization and surgical planning.

Surgical technique

Group A: TBW

Patients allocated to Group A were treated using the TBW technique. Surgery was performed under spinal anesthesia with the patient in the supine position. A longitudinal midline incision was made over the anterior aspect of the knee to expose the fracture site. After achieving anatomical reduction using pointed reduction forceps under fluoroscopic guidance, two parallel 2 mm Kirschner wires (K-wires) were introduced longitudinally across the fracture. Once satisfactory reduction is obtained and maintained, two parallel 2.0 mm K-wires are introduced longitudinally across the fracture line, from the inferior pole to the superior pole of the patella (or vice versa, depending on surgeon preference). The K-wires are placed in the anterior half of the patella, parallel to each other and perpendicular to the fracture plane. This positioning is critical to provide optimal purchase in both fragments while avoiding penetration of the articular surface.

The wires act as guide and anchor elements for the tension band construct. Their parallel placement ensures uniform load sharing and prevents rotational instability of the fracture fragments. Accurate depth control is essential; the K-wires should engage the opposite fragment securely without protruding into the patellofemoral joint, which is confirmed fluoroscopically. An 18-gauge stainless steel wire was then applied in a vertical figure-of-eight configuration close to the bone-wire interface to convert tensile forces into compressive forces at the fracture site. The construct was tensioned adequately, the proximal ends of the K-wires were bent and embedded within the quadriceps tendon, and the distal ends were trimmed flush to reduce soft-tissue irritation.

Group B: Star Plate Fixation

**Figure 2:**
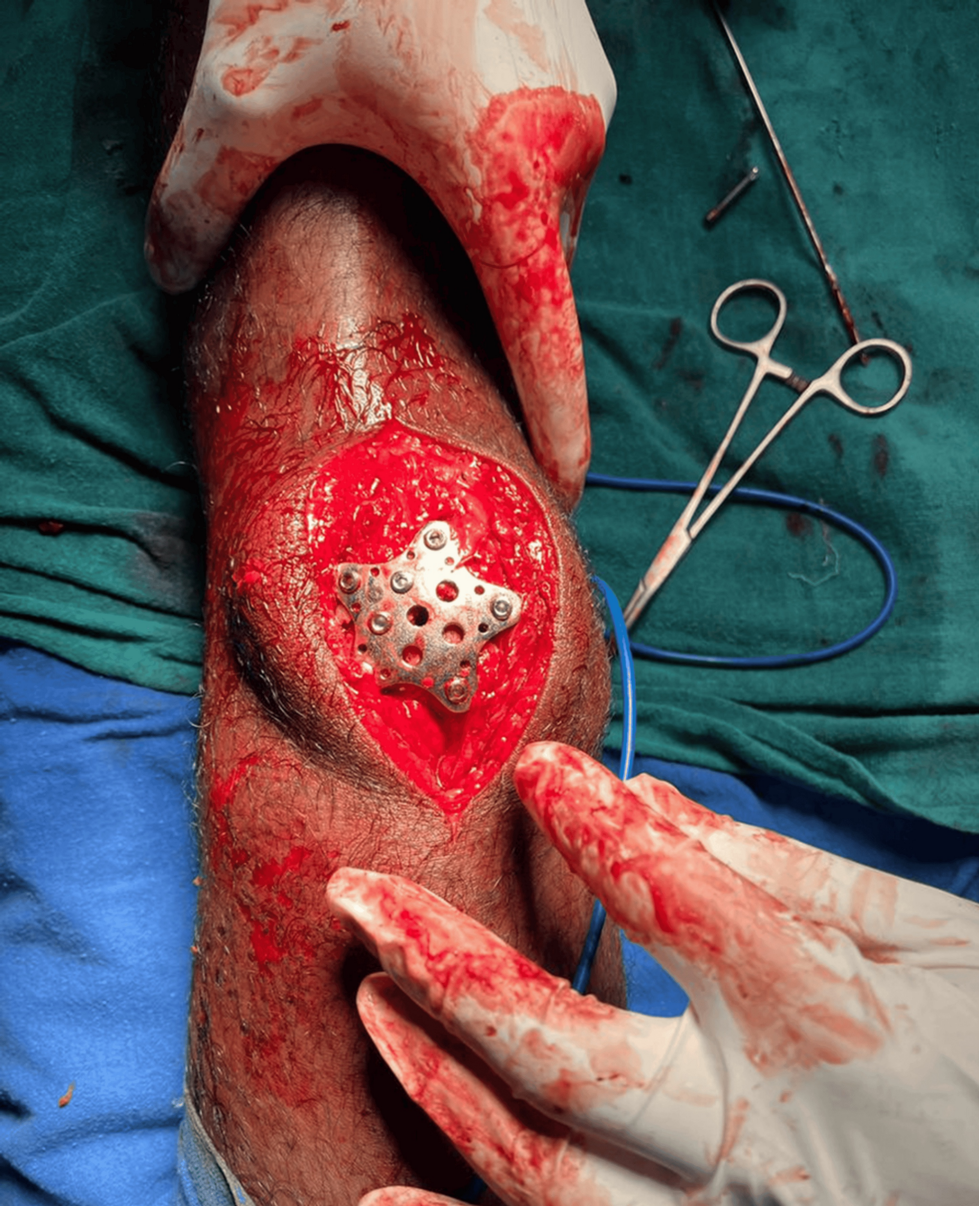
Intraoperative image of star plate patella fixation in a transverse patella fracture. Source: Image courtesy of the authors.

In Group B, fracture exposure and reduction were performed using a similar surgical approach. Following anatomical reduction, fixation was achieved using a precontoured star-shaped patellar plate placed on the anterior surface of the patella. Multiple locking screws were inserted through the plate arms into the proximal and distal fragments to provide stable fixation. Fluoroscopic imaging was used to confirm satisfactory reduction and appropriate implant positioning (Figure 2).

Postoperative management

Postoperatively, all patients received uniform care, including antibiotic coverage, analgesics, and thromboprophylaxis. Isometric quadriceps strengthening exercises were initiated on the first postoperative day. Patients were encouraged to begin knee mobilization and full weight-bearing as tolerated. Wound inspection was performed on the second postoperative day, and sutures were removed between postoperative days 10 and 12.

Follow-up and outcome assessment

Patients were reviewed at two, four, eight, and 12 weeks following surgery and subsequently on a monthly basis up to six months. Clinical assessment during follow-up included evaluation of pain, knee range of motion, extensor lag, quadriceps strength, and implant-related complications. Radiographic assessment was conducted at each visit to monitor fracture healing, implant position, and any evidence of loss of reduction.

Functional outcome was evaluated using the Böstman scoring system as originally described by Böstman et al. for the assessment of clinical outcomes following patellar fractures [3]. Fracture union was defined radiologically by the presence of cortical continuity across the fracture site and clinically by the absence of localized tenderness.

Statistical analysis

All collected data were entered into Microsoft Excel (Microsoft Corp., Redmond, WA, USA) and analyzed using Statistical Package for the Social Sciences (SPSS) software (IBM Corp., Armonk, NY, USA). Categorical variables were presented as frequencies and percentages, while continuous variables were expressed as mean ± standard deviation. Intergroup comparisons of categorical variables were performed using the Chi-squared (χ²) test, and continuous variables were analyzed using Student’s t-test. A p-value of less than 0.05 was considered statistically significant.

## Results

Demographic and injury characteristics

The study included 40 patients diagnosed with displaced transverse fractures of the patella, with 20 patients assigned to each treatment group: Group A (TBW) and Group B (star plate fixation). In Group A, 12 patients (60%) were between 18 and 40 years of age, while eight patients (40%) were aged 41-65 years. In Group B, 11 patients (55%) were in the younger age group, and nine patients (45%) belonged to the older age group. Statistical analysis showed no significant difference in age distribution between the two groups (χ² = 0.10, p = 0.75).

Male patients constituted the majority in both cohorts, with 15 men (75%) in Group A and 12 men (60%) in Group B. The difference in gender distribution between the two groups was not statistically significant (χ² = 1.03, p = 0.31).

Road traffic accidents represented the most frequent mechanism of injury in both groups, accounting for 10 cases (50%) in the TBW group and 11 cases (55%) in the star plate group. Other mechanisms included falls from height and miscellaneous causes. The distribution of injury mechanisms was comparable between the groups, with no statistically significant difference observed (χ² = 0.62, p = 0.73).

Laterality analysis revealed a higher incidence of right-sided knee involvement in both groups. In Group A, the right knee was affected in 13 patients (65%), while in Group B, 15 patients (75%) sustained right-sided injuries. This difference was not statistically significant (χ² = 0.48, p = 0.49). The detailed demographic and injury-related data are summarized in Table 1.

**Table 1:** Demographic data of patients having a transverse patella fracture.

Variable		Group A		Group B		t-score	p-value
		Number	Percentage	Number	Percentage		
Age group (years)	18 to 40	12	60	11	55	0.1	0.75
	41 to 65	8	40	9	45		
Gender	Male	15	75	12	60	1.03	0.5
	Female	5	25	8	40		
Etiology	Road traffic accident	10	50	11	55	0.62	0.73
	Fall from height	7	35	5	25		
	Others	3	15	4	20		
Side of injury	Right side	13	65	15	75	0.48	0.49
	Left side	7	35	5	25		

Fracture union and radiological outcomes

Radiographic evidence of fracture healing was observed in all patients treated with TBW (20/20; 100%) and in 19 patients (95%) who underwent star plate fixation, with one patient in Group B developing non-union. Comparison of union rates between the two groups showed no statistically significant difference (χ² = 1.03, p = 0.31).

The average duration to radiological union was 10.9 ± 1.3 weeks in Group A and 11.9 ± 1.5 weeks in Group B. Statistical analysis using an independent samples t-test revealed no significant difference in time to union between the two fixation techniques (t = 0.97, p = 0.338). Detailed radiological outcomes are presented in Tables 2, 3.

**Table 2:** Fracture union rate in patients who underwent tension band wiring vs. star plate patella fixation.

Fracture union rate	Group A		Group B			
	Number	Percentage	Number	Percentage	t-score	p-value
Union	20	20	19	95	1.03	0.31
Non-union	0	0	1	5		
Total	20	100	20	100		

**Table 3:** Time to radiological union of patients who underwent tension band wiring vs. star plate patella fixation. SD: standard deviation

Radiological union (weeks)	Group A	Group B
Mean	10.9	11.9
SD	1.3	1.5
t-value	0.97	
p-value	0.338	

Functional outcomes (Böstman score)

**Figure 3:**
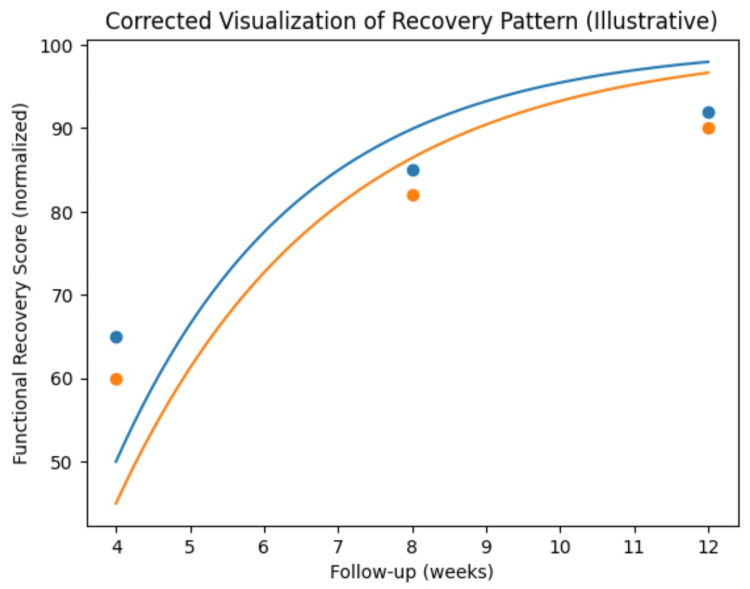
Comparison using the Böstman score during the follow-up of patients who underwent tension band wiring vs. star plate patella fixation.

Functional evaluation using the Böstman scoring system demonstrated excellent results in 15 patients (75%) and good results in five patients (25%) in the TBW group. In the star plate group, excellent outcomes were achieved in 14 patients (70%), good outcomes in four patients (20%), and satisfactory outcomes in two patients (10%). No patient in either group demonstrated a poor functional outcome (Figure 3).

Statistical comparison of functional outcomes between the two groups showed no significant difference (χ² = 0.42, p = 0.8116). Functional assessment was performed using the Böstman scoring system as originally described by Böstman et al. [3]. A summary of functional outcomes is provided in Table 4.

**Table 4:** Functional outcome of patients who underwent tension band wiring vs. star plate patella fixation using the Böstman scoring system.

Outcome	Group A		Group B	
	Number	Percentage	Number	Percentage
Excellent (28 to 30)	15	75	14	70
Good (20 to 27)	5	25	4	20
Satisfactory (<20)	0	0	2	10
Total	20	100	20	100
t-value	0.42			
p-value	0.8116			

Postoperative clinical progress and follow-up findings

Pain and Range of Motion

At the two-week postoperative assessment, the majority of patients in both groups reported moderate anterior knee discomfort, which showed progressive improvement over subsequent follow-up visits. By 8-12 weeks, pain levels had significantly decreased, and by six months, most patients were pain-free regardless of the fixation method used.

Knee mobility improved steadily over time in both cohorts. At four weeks postoperatively, mean knee flexion ranged between 70° and 80°, which increased to more than 110° by 12 weeks. Near-normal knee range of motion was achieved in most patients by six months, with no statistically significant difference between the two groups.

Extensor Lag and Quadriceps Strength

A mild extensor lag was observed in a limited number of patients during early follow-up, particularly between two and four weeks postoperatively, and was more frequently noted in the star plate group. With continued physiotherapy, extensor lag resolved by 12 weeks in all affected patients. Progressive improvement in quadriceps strength was noted, with most patients attaining near-normal muscle power (Grades 4-5) by the final follow-up at six months.

Radiographic Evaluation

**Figure 4:**
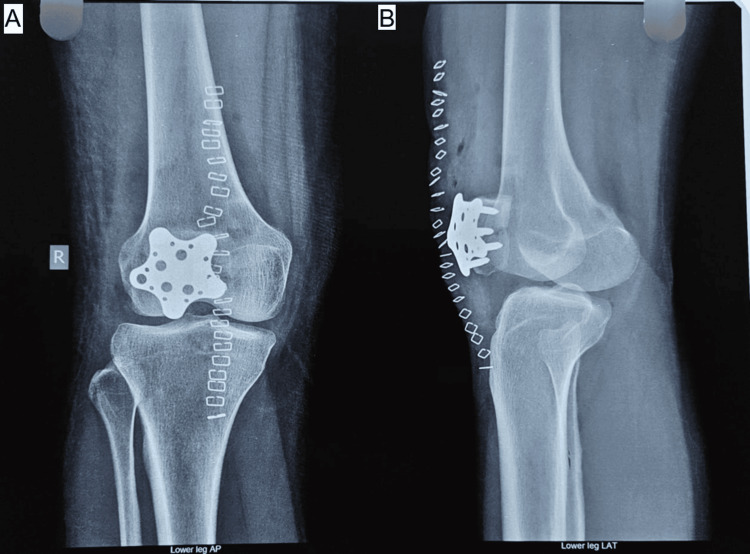
(A, B) Postoperative radiograph of star plate patella fixation in a transverse patella fracture at 4 weeks. Source: Images courtesy of the authors.

**Figure 5:**
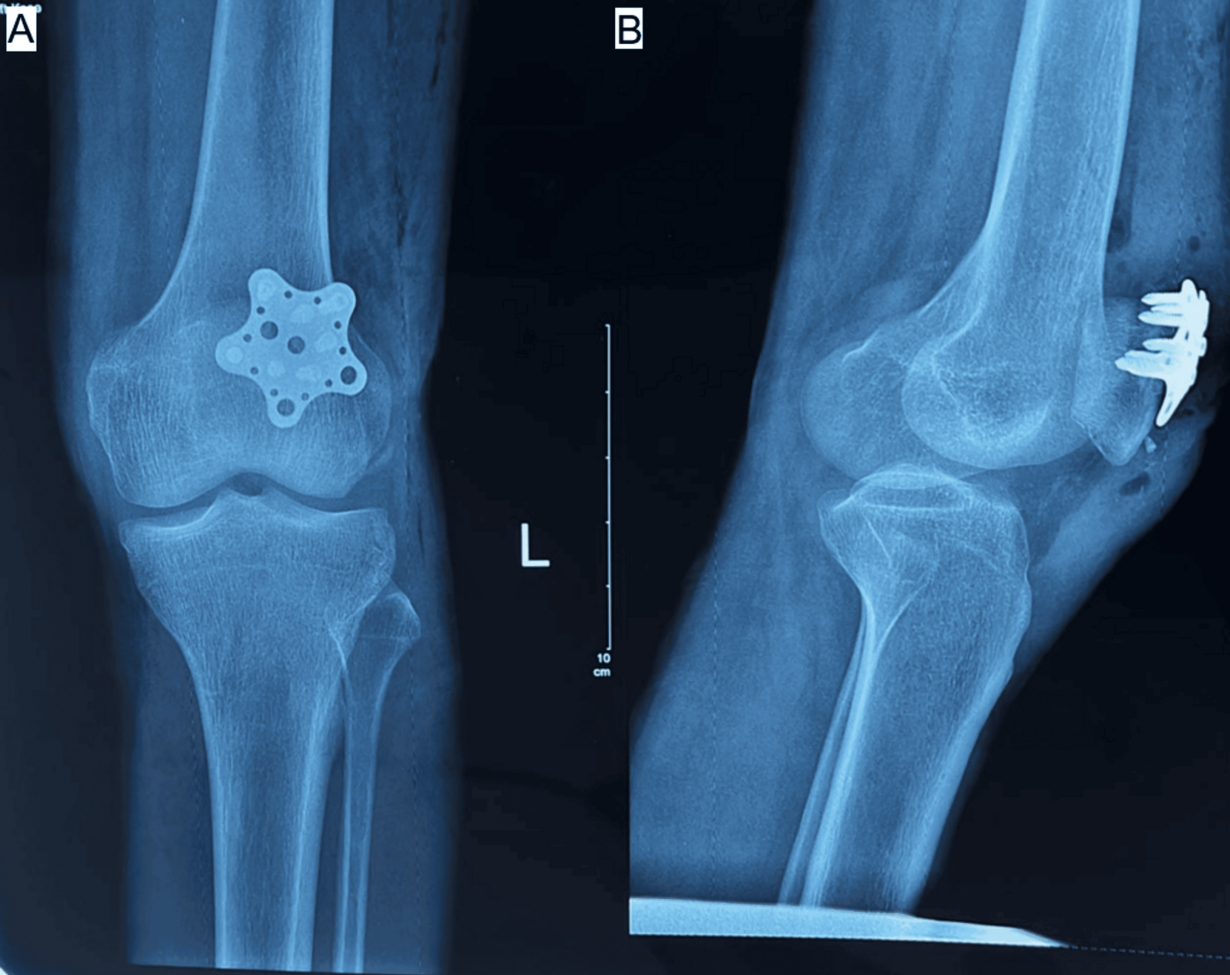
(A, B) Postoperative radiograph of star plate patella fixation in transverse patella fractures at 12 weeks. Source: Images courtesy of the authors.

**Figure 6:**
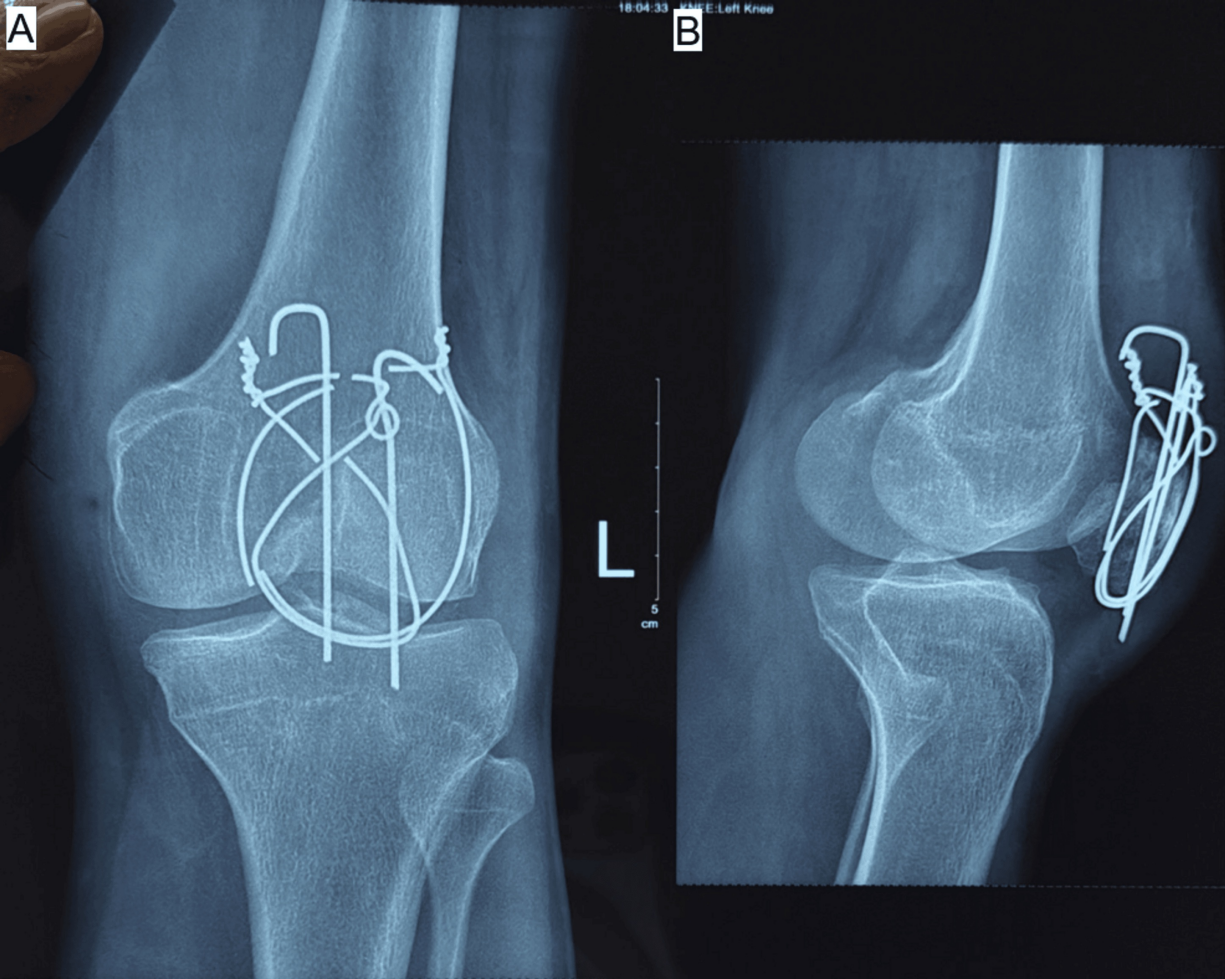
(A, B) Postoperative radiograph of TBW patella in transverse patella fracture at 6 months. Source: Images courtesy of the authors. TBW: tension band wiring

**Figure 7:**
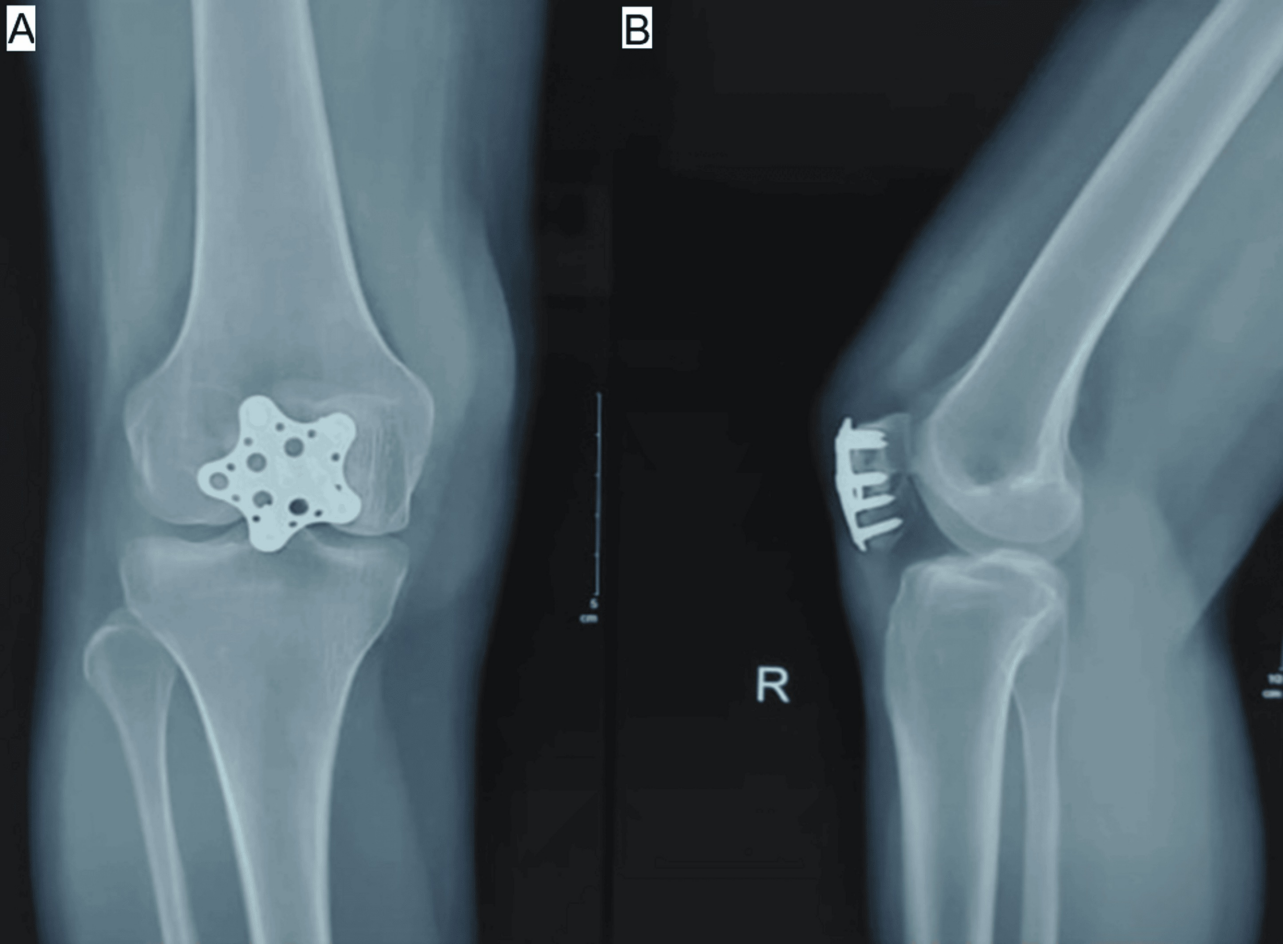
(A, B) Postoperative radiograph of star plate patella fixation in a transverse patella fracture at 6 months. Source: Images courtesy of the authors.

Sequential radiographic assessments, including anteroposterior, lateral, and skyline views obtained at two, four, eight, and 12 weeks and at monthly intervals thereafter, demonstrated consistent progression toward fracture healing in both groups. Implant positioning remained satisfactory throughout follow-up in all but one patient in Group B, who developed non-union. No cases of implant failure, breakage, or migration were observed (Figures 4-7).

Complications

The overall incidence of postoperative complications was low in both groups. In the TBW group, one patient (5%) developed a superficial surgical site infection, and one patient (5%) experienced knee stiffness. In the star plate fixation group, two patients (10%) developed superficial infections, two patients (10%) experienced knee stiffness, and one patient (5%) developed non-union. Although the complication rate was marginally higher in the star plate group, both fixation techniques demonstrated comparable fracture union times and functional outcomes. A detailed summary of complications is presented in Table 5.

**Table 5:** Complications.

Complications	Group A		Group B			
	Number	Percentage	Number	Percentage	t-value	p-value
Superficial infection	1	5	2	10	0.36	0.55
Knee stiffness	1	5	2	10		
Non-union	0	0	1	5		

## Discussion

Recent epidemiological studies report an annual incidence of patellar fractures of approximately 13.1 per 100,000 individuals, accounting for nearly 0.5% of all skeletal injuries. The principal objective in treating these fractures is restoration of the knee extensor mechanism and preservation of patellofemoral articular congruity, as impairment of either can result in substantial functional limitation [7]. Management decisions are primarily influenced by fracture morphology, degree of displacement, and integrity of the extensor mechanism. Fractures with minimal displacement and preserved extensor function, typically defined by articular incongruity of less than 2-3 mm, are often suitable for non-operative management using immobilization followed by early rehabilitation [8]. In contrast, fractures with significant displacement, comminution, or disruption of extensor continuity generally necessitate surgical intervention to achieve anatomical reduction and stable fixation.

Among the various operative techniques available, TBW-commonly combined with K-wires or cannulated screws-continues to be widely employed and is regarded as the standard treatment for transverse patellar fractures in many orthopedic centers. This technique functions by transforming tensile forces acting across the anterior patella into compressive forces at the fracture site, thereby facilitating fracture union while allowing early knee mobilization [9-11]. In light of emerging fixation options, the present study was undertaken to compare the clinical and radiological effectiveness of TBW with star plate fixation in patients presenting with displaced transverse patellar fractures. With the availability of various government schemes, expensive star plate patella implants have been made available free of cost and have henceforth increased demand in the population. Therefore, we applied a star plate patella in transverse fractures, though not indicated, to measure the functional outcome over TBW. Moreover, our institute observed greater hardware irritation and migration with TBW, whereas these complications were less frequent with the star plate patella when proper repair of subcutaneous tissue was performed intraoperatively.

In the current series, the demographic distribution was comparable between the two treatment groups. In the TBW group, 60% of patients were between 18 and 40 years of age, while 40% were aged 41-65 years; corresponding values in the star plate group were 55% and 45%, respectively. Radiological union was achieved in all patients treated with TBW and in 95% of patients managed with star plate fixation, with one case of non-union in the latter group. The mean time to fracture union was 10.9 weeks in Group A and 11.9 weeks in Group B, and this difference was not statistically significant (p = 0.338). These findings are consistent with previous clinical reports supporting the reliability of TBW. Javali reported complete fracture union in a retrospective series of 21 patients with transverse patellar fractures treated using TBW, with a mean Böstman score of 26.54 and predominantly excellent or good functional outcomes [12].

Biomechanical evidence has suggested potential advantages of locking plate constructs over conventional TBW. Warner et al. compared lateral rim variable-angle locking plates with TBW in cadaveric models of both simple and complex patellar fractures and demonstrated significantly reduced articular displacement and fragment rotation in the locking plate group during 5,000 simulated loading cycles, indicating superior construct stability under dynamic conditions [13].

Functional outcomes in this study were quantified using the Böstman scoring system, a validated clinical assessment tool for patellar fracture outcomes originally proposed by Böstman et al. [3], which were comparable between the two groups. Excellent outcomes were observed in 75% of patients treated with TBW and in 70% of patients who underwent star plate fixation, while the remaining patients achieved good or satisfactory results. No statistically significant difference in functional outcome was identified between the groups (p = 0.8116). The overall complication rate was low, although slightly higher in the star plate group, which included a greater incidence of superficial infection, knee stiffness, and one case of non-union. In the present study, the occurrence of non-union exclusively in the star plate fixation group warrants focused biomechanical interpretation.

TBW functions on the principle of dynamic compression, whereby tensile forces generated by the quadriceps during knee flexion are converted into compressive forces at the fracture interface, promoting progressive fracture gap closure and osteosynthesis. This mechanism is particularly effective in simple transverse fracture patterns and is the primary reason TBW remains the biomechanical gold standard for such fractures.

In contrast, star plate constructs provide rigid, load-sharing fixation without dynamic compression. When applied to a simple transverse fracture, the plate may act as a stress-shielding device, reducing physiologic load transfer across the fracture site. Additionally, plate contouring and screw fixation can inadvertently maintain a static fracture gap or even produce subtle distraction, particularly on the anterior surface of the patella, thereby inhibiting interfragmentary compression. This combination of stress shielding and distraction at the fracture interface offers a plausible biomechanical explanation for the non-union observed in the star plate group.

Comparable results have been reported in studies evaluating locking plate fixation for patellar fractures. Singh et al. assessed clinical outcomes in patients with acute patellar fractures treated with unidirectional angle-fixed low-profile titanium locking plates and reported fracture union in 19 of 20 patients, with excellent postoperative knee motion and no residual extensor lag at final follow-up [14]. Similarly, Stoffel et al. demonstrated that anterior variable-angle locking plates provided significantly greater resistance to displacement and fragment rotation than TBW in both simple and complex fracture patterns under prolonged cyclic loading, reinforcing the biomechanical superiority of locked plating constructs (p ≤ 0.01) [15]. The incision length for the star plate patella is the same as that for TBW of the patella, with a maximum of 8 cm. There was no issue of cosmesis in the results. The postoperative scar length was the same in both techniques.

## Conclusions

Both TBW and star plate fixation produced satisfactory clinical and radiological outcomes in the treatment of displaced transverse patellar fractures, with comparable rates of fracture union, time to healing, and functional recovery as measured by the Böstman scoring system. Star plate fixation offered no functional benefit and was associated with a clinically important complication in transverse patellar fractures. The findings reinforce that TBW remains the preferred and biomechanically appropriate fixation method for this fracture pattern, while star plates should be reserved for comminuted fractures where dynamic compression is not achievable. Star plate fixation was associated with higher complication rates, including non-union, without demonstrating superior functional outcomes compared to TBW.

Serial clinical and radiographic evaluations showed consistent improvement in pain relief, knee range of motion, quadriceps strength, and extensor mechanism function in both treatment groups, with no significant differences observed at the final follow-up. These findings indicate that TBW remains a dependable and cost-effective option for simple transverse patellar fractures, while star plate fixation may be an alternative in comminuted patellar fractures, where tension band principles are not applicable; however, such fractures were explicitly excluded from the present study. The choice of fixation technique should ultimately be guided by fracture characteristics, soft-tissue status, surgeon experience, and individual patient factors to achieve optimal outcomes.
